# Supplementary material for: Transcriptome Analysis Reveals Vimentin-Induced Disruption of Cell–Cell Associations Augments Breast Cancer Cell Migration
Source: Cells. 2022 Dec 13;11(24):4035. doi: 10.3390/cells11244035 (PMC9776984; doi:10.3390/cells11244035)
Supplement: Supplementary file 1 [file cells-11-04035-s001.zip › DEGs and enrichment analysis.zip!/DEGs and enrichment analysis/WT_VIMvsVector GO enrichment/GSEA/WT_VIM/POLYSOME/POLYSOME(GO_0005844).html]

Details for gene set POLYSOME(GO:0005844)[GSEA]


|  || Dataset | fpkm.sample |
| Phenotype | sample.cls |
| Upregulated in class | WT\_VIM |
| GeneSet | POLYSOME(GO:0005844) |
| Enrichment Score (ES) | 0.9110147 |
| Normalized Enrichment Score (NES) | 1.462361 |
| Nominal p-value | 0.0 |
| FDR q-value | 0.16299984 |
| FWER p-Value | 0.163 |
Table: GSEA Results Summary

  

Fig 1: Enrichment plot: POLYSOME(GO:0005844)
  
Profile of the Running ES Score & Positions of GeneSet Members on the Rank Ordered List

  

| PROBE | DESCRIPTION   (from dataset) | GENE SYMBOL | GENE\_TITLE | RANK IN GENE LIST | RANK METRIC SCORE | RUNNING ES | CORE ENRICHMENT || 1 | ENSG00000026025 | VIM |  |  | 2388 | 1709.539 | 0.9110 | Yes |
| 2 | ENSG00000171858 | RPS21 |  |  | 3544 | 3.623 | 0.8935 | No |
| 3 | ENSG00000183011 | NAA38 |  |  | 3974 | 3.055 | 0.8879 | No |
| 4 | ENSG00000213741 | RPS29 |  |  | 4449 | 2.684 | 0.8814 | No |
| 5 | ENSG00000161016 | RPL8 |  |  | 4659 | 2.582 | 0.8793 | No |
| 6 | ENSG00000105640 | RPL18A |  |  | 5448 | 2.217 | 0.8672 | No |
| 7 | ENSG00000167658 | EEF2 |  |  | 5501 | 2.199 | 0.8675 | No |
| 8 | ENSG00000233927 | RPS28 |  |  | 5826 | 2.087 | 0.8632 | No |
| 9 | ENSG00000130255 | RPL36 |  |  | 6038 | 2.023 | 0.8607 | No |
| 10 | ENSG00000187778 | MCRS1 |  |  | 6107 | 2.002 | 0.8607 | No |
| 11 | ENSG00000179218 | CALR |  |  | 6278 | 1.979 | 0.8589 | No |
| 12 | ENSG00000089280 | FUS |  |  | 6690 | 1.880 | 0.8530 | No |
| 13 | ENSG00000149273 | RPS3 |  |  | 7575 | 1.700 | 0.8390 | No |
| 14 | ENSG00000128829 | EIF2AK4 |  |  | 7686 | 1.680 | 0.8381 | No |
| 15 | ENSG00000148303 | RPL7A |  |  | 7776 | 1.661 | 0.8375 | No |
| 16 | ENSG00000063177 | RPL18 |  |  | 7924 | 1.637 | 0.8359 | No |
| 17 | ENSG00000142676 | RPL11 |  |  | 8542 | 1.543 | 0.8263 | No |
| 18 | ENSG00000198034 | RPS4X |  |  | 8797 | 1.507 | 0.8229 | No |
| 19 | ENSG00000185721 | DRG1 |  |  | 8947 | 1.489 | 0.8212 | No |
| 20 | ENSG00000197728 | RPS26 |  |  | 8984 | 1.483 | 0.8214 | No |
| 21 | ENSG00000108298 | RPL19 |  |  | 9003 | 1.480 | 0.8219 | No |
| 22 | ENSG00000114867 | EIF4G1 |  |  | 9408 | 1.430 | 0.8159 | No |
| 23 | ENSG00000198755 | RPL10A |  |  | 9621 | 1.402 | 0.8130 | No |
| 24 | ENSG00000229117 | RPL41 |  |  | 9683 | 1.394 | 0.8128 | No |
| 25 | ENSG00000106682 | EIF4H |  |  | 9945 | 1.365 | 0.8091 | No |
| 26 | ENSG00000144713 | RPL32 |  |  | 10364 | 1.319 | 0.8028 | No |
| 27 | ENSG00000197181 | PIWIL2 |  |  | 10472 | 1.318 | 0.8017 | No |
| 28 | ENSG00000172809 | RPL38 |  |  | 10514 | 1.316 | 0.8017 | No |
| 29 | ENSG00000089154 | GCN1 |  |  | 10528 | 1.314 | 0.8023 | No |
| 30 | ENSG00000071082 | RPL31 |  |  | 10803 | 1.283 | 0.7983 | No |
| 31 | ENSG00000132478 | UNK |  |  | 10895 | 1.275 | 0.7975 | No |
| 32 | ENSG00000144535 | DIS3L2 |  |  | 11181 | 1.249 | 0.7934 | No |
| 33 | ENSG00000156482 | RPL30 |  |  | 11221 | 1.245 | 0.7934 | No |
| 34 | ENSG00000186468 | RPS23 |  |  | 11772 | 1.204 | 0.7848 | No |
| 35 | ENSG00000155506 | LARP1 |  |  | 11962 | 1.185 | 0.7822 | No |
| 36 | ENSG00000135829 | DHX9 |  |  | 12040 | 1.178 | 0.7816 | No |
| 37 | ENSG00000060138 | YBX3 |  |  | 12113 | 1.172 | 0.7810 | No |
| 38 | ENSG00000137154 | RPS6 |  |  | 12419 | 1.148 | 0.7765 | No |
| 39 | ENSG00000138035 | PNPT1 |  |  | 12633 | 1.129 | 0.7735 | No |
| 40 | ENSG00000129245 | FXR2 |  |  | 12972 | 1.103 | 0.7684 | No |
| 41 | ENSG00000111832 | RWDD1 |  |  | 12982 | 1.103 | 0.7689 | No |
| 42 | ENSG00000063046 | EIF4B |  |  | 13256 | 1.082 | 0.7648 | No |
| 43 | ENSG00000134001 | EIF2S1 |  |  | 13503 | 1.063 | 0.7613 | No |
| 44 | ENSG00000092847 | AGO1 |  |  | 13767 | 1.044 | 0.7574 | No |
| 45 | ENSG00000123908 | AGO2 |  |  | 14056 | 1.021 | 0.7531 | No |
| 46 | ENSG00000155959 | VBP1 |  |  | 14247 | 1.007 | 0.7504 | No |
| 47 | ENSG00000135097 | MSI1 |  |  | 14388 | 0.996 | 0.7486 | No |
| 48 | ENSG00000129084 | PSMA1 |  |  | 15301 | 0.952 | 0.7337 | No |
| 49 | ENSG00000114391 | RPL24 |  |  | 15304 | 0.952 | 0.7342 | No |
| 50 | ENSG00000100902 | PSMA6 |  |  | 15339 | 0.950 | 0.7342 | No |
| 51 | ENSG00000198918 | RPL39 |  |  | 16613 | 0.881 | 0.7131 | No |
| 52 | ENSG00000089009 | RPL6 |  |  | 16873 | 0.865 | 0.7092 | No |
| 53 | ENSG00000133026 | MYH10 |  |  | 17001 | 0.857 | 0.7075 | No |
| 54 | ENSG00000107929 | LARP4B |  |  | 17180 | 0.846 | 0.7050 | No |
| 55 | ENSG00000241343 | RPL36A |  |  | 18026 | 0.798 | 0.6911 | No |
| 56 | ENSG00000135040 | NAA35 |  |  | 18104 | 0.793 | 0.6902 | No |
| 57 | ENSG00000112425 | EPM2A |  |  | 18358 | 0.778 | 0.6864 | No |
| 58 | ENSG00000153944 | MSI2 |  |  | 18782 | 0.754 | 0.6797 | No |
| 59 | ENSG00000166173 | LARP6 |  |  | 18785 | 0.754 | 0.6800 | No |
| 60 | ENSG00000154059 | IMPACT |  |  | 18923 | 0.747 | 0.6781 | No |
| 61 | ENSG00000139977 | NAA30 |  |  | 19132 | 0.735 | 0.6750 | No |
| 62 | ENSG00000114416 | FXR1 |  |  | 19154 | 0.734 | 0.6751 | No |
| 63 | ENSG00000151461 | UPF2 |  |  | 20261 | 0.673 | 0.6567 | No |
| 64 | ENSG00000102081 | FMR1 |  |  | 21803 | 0.589 | 0.6310 | No |
| 65 | ENSG00000204842 | ATXN2 |  |  | 21859 | 0.585 | 0.6304 | No |
| 66 | ENSG00000161813 | LARP4 |  |  | 22229 | 0.562 | 0.6244 | No |
| 67 | ENSG00000108256 | NUFIP2 |  |  | 24065 | 0.432 | 0.5936 | No |
| 68 | ENSG00000131914 | LIN28A |  |  | 27066 | 0.097 | 0.5429 | No |
| 69 | ENSG00000125207 | PIWIL1 |  |  | 32509 | NaN | 0.4507 | No |
| 70 | ENSG00000169297 | NR0B1 |  |  | 40712 | NaN | 0.3119 | No |
| 71 | ENSG00000129824 | RPS4Y1 |  |  | 42014 | NaN | 0.2898 | No |
| 72 | ENSG00000165496 | RPL10L |  |  | 42573 | NaN | 0.2804 | No |
| 73 | ENSG00000092345 | DAZL |  |  | 45762 | NaN | 0.2264 | No |
Table: GSEA details
[plain text format]

  

Fig 2: POLYSOME(GO:0005844)
  
Blue-Pink O' Gram in the Space of the Analyzed GeneSet

  

Fig 3: POLYSOME(GO:0005844): Random ES distribution
  
Gene set null distribution of ES for
 **POLYSOME(GO:0005844)**
